# Urinary polycyclic aromatic hydrocarbon metabolites and hyperlipidemia: NHANES 2007–2016

**DOI:** 10.1186/s12944-024-02153-6

**Published:** 2024-05-27

**Authors:** Chenle Ye, Yuanrun Liu, Zhuoqi He, Weikai Huang, Guangzhan Chen, Tieli Peng, Kaishu Li

**Affiliations:** 1 Division of Gastroenterology, Institute of Digestive Disease of Guangzhou Medical University, Affiliated Qingyuan Hospital, Guangzhou Medical University, Qingyuan People’s Hospital, Qingyuan, 511518 China; 2 Department of Neurosurgery, Affiliated Qingyuan Hospital, Guangzhou Medical University, Qingyuan People’s Hospital, Qingyuan, 511518 China; 3 Institute of Digestive Disease of Guangzhou Medical University, Affiliated Qingyuan Hospital, Guangzhou Medical University, Qingyuan People’s Hospital, Qingyuan, 511518 China

**Keywords:** Hyperlipidemia, Polycyclic aromatic hydrocarbons, Urinary PAH metabolites, Cross-sectional study

## Abstract

**Background:**

The relationships between urinary polycyclic aromatic hydrocarbon (PAH) metabolites and hyperlipidemia have not been thoroughly studied. The primary goal of this research focused on investigating the linkage between PAH metabolite concentrations in urine and hyperlipidemia prevalence within US adults.

**Methods:**

A cross-sectional analysis was conducted using data from the 2007–2016 National Health and Nutrition Examination Survey (NHANES). Logistic regression models were used to assess correlations between urinary PAH metabolite levels and the risk of hyperlipidemia, while restricted cubic spline models were used to examine dose‒response relationships. Subgroup and interaction analyses were performed to further elucidate these associations. Weighted quantile sum (WQS) regression analyzed the cumulative impact of various urinary PAH metabolites on hyperlipidemia risk.

**Results:**

This study included 7,030 participants. Notably, individuals in the highest quintile of urinary PAH metabolite concentrations exhibited a significantly elevated prevalence of hyperlipidemia, even after comprehensive adjustments (odds ratio [OR]: 1.33, 95% confidence interval [CI]: 1.01–1.75). Moreover, elevated levels of 1-hydroxyphenanthrene and 2-hydroxynaphthalene in the fourth quintile and 2-hydroxyfluorene in the third, fourth, and fifth quintiles demonstrated positive correlations with the prevalence of hyperlipidemia. These associations persisted across subgroup analyses. Additionally, a positive correlation between the urinary PAH metabolite mixture and hyperlipidemia (positive model: OR = 1.04, 95% CI: 1.00-1.09) was observed in the WQS model, and 2-hydroxynaphthalene showed the most substantial contribution.

**Conclusion:**

The cross-sectional analysis identified a significant correlation between urinary PAH metabolite and hyperlipidemia prevalence within the US demographic, with 2-hydroxynaphthalene being the predominant influencer. These findings underscore the need to mitigate PAH exposure as a preventive measure for hyperlipidemia.

**Supplementary Information:**

The online version contains supplementary material available at 10.1186/s12944-024-02153-6.

## Introduction

Hyperlipidemia, a systemic metabolic disorder, manifests through elevated plasma levels of one or more lipid fractions beyond normative thresholds, affecting approximately 10% of adults [1]. This disorder predominantly manifests as heightened low-density lipoprotein cholesterol (LDL-C) and levels of plasma triglycerides (TGs), which are pivotal contributors to a spectrum of medical conditions, including diabetes mellitus, hypertension, obesity, and nonalcoholic fatty liver disease [2, 3]. These conditions significantly exacerbate risks to human health. Studies in epidemiology have identified hyperlipidemia as a critical determinant in the development of atherosclerosis, which in turn heightens the risk for coronary artery disease, myocardial infarction, cerebrovascular accidents, and a spectrum of cardiovascular diseases [4]. In the US alone, an estimated 28 million individuals exhibit cholesterol levels exceeding 240 mg/dL [5]. In 2019, approximately 8.54 million deaths worldwide were attributed to ischemic heart disease, with high plasma LDL-C levels being implicated in approximately 3.78 million of these cases [3]. Concurrently, ischemic stroke caused approximately 2.73 million deaths, with 610,000 fatalities linked to elevated plasma LDL-C concentrations [3]. The etiology of hyperlipidemia is multifactorial, encompassing a plethora of genetic variants and environmental determinants such as pollutants, smoking, alcohol intake, dietary indiscretion, and a sedentary lifestyle, with an increasing focus on the impact of environmental factors on lipid profiles [6].

Among environmental pollutants, polycyclic aromatic hydrocarbons (PAHs) are prevalent contaminants originating from the incomplete combustion of organic matter, including oil, tobacco, and fossil fuels, as well as food preparation processes [7]. To date, more than 200 PAH compounds have been identified, including naphthalene, phenanthrene, anthracene, and pyrene [8]. Human exposure to PAHs predominantly occurs through inhalation, ingestion, dermal contact, and transplacental transfer [9]. PAHs are potential teratogens, carcinogens, and mutagens [7]. Their metabolites, which are water-soluble, are usually eliminated through feces and urine. Mono-hydroxylated polycyclic aromatic hydrocarbons (OH-PAHs) act as indicators for PAH presence in humans [10, 11]. Associations between PAH exposure and chronic diseases, such as cardiovascular disease [12], diabetes [13], and cancer [14, 15], have been documented, underscoring the significant health risks posed by these compounds.

Despite the increasing volume of epidemiological research exploring the effects of PAH exposure on lipid metabolism, findings remain inconsistent [16–19]. For instance, Ma et al. observed a positive correlation, demonstrating that elevated levels of PAH metabolites in urine are associated with an increase in total cholesterol (TC) and LDL-C concentrations [16]. In contrast, research conducted by Hu et al. revealed a negative correlation between urinary PAH metabolites and TGs levels [19]. The link between urinary PAH metabolites and hyperlipidemia in the broader population remains unclear. This study hypothesized that higher urinary PAH metabolite levels correlate with an increased risk of hyperlipidemia. By examining these relationships, the study aims to pinpoint potential environmental hazards and contribute to the formulation of public health policies and personal wellness management strategies, ultimately aiming to decrease the prevalence of hyperlipidemia and its associated conditions. This investigation examined data from the 2007–2016 National Health and Nutrition Examination Survey (NHANES) to delineate the association between PAH exposure, which was quantified through urinary PAH metabolites, and hyperlipidemia among US adults. Additionally, this study explored the combined effects of urinary PAH metabolite mixtures on hyperlipidemia.

### Purpose of the current study

The current study aimed to explore relationships between urinary PAH metabolite levels and hyperlipidemia prevalence in a representative population sample to fill knowledge gaps in this area from previous studies. Elucidating these potential relationships may help clarify the health effects of environmental pollutants and contribute to hyperlipidemia prevention at the public health level.

## Methods

### Study cohort

Conducted by the National Center for Health Statistics (NCHS) within the Centers for Disease Control and Prevention (CDC), NHANES represents a comprehensive, nationally representative research initiative within the US. This survey amalgamates household interviews with standardized medical evaluations conducted at a mobile examination center, including both physical assessments and laboratory investigations. Participant selection was executed via an intricate, multistage, probability sampling technique, ensuring a representative cross-section of the US civilian, noninstitutionalized population. Ethical approval for all study procedures was granted by the Ethics Review Board of the NCHS, with participants providing written informed consent prior to data collection.

**Fig. 1:**
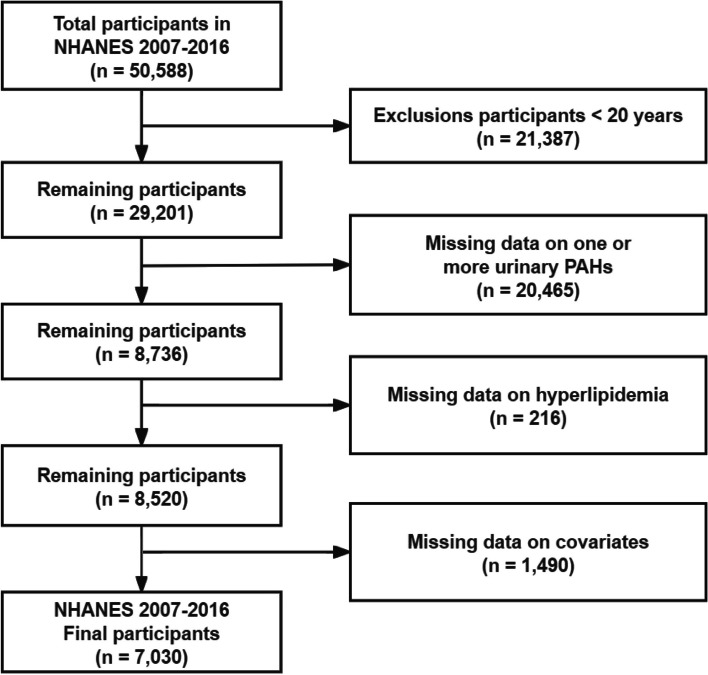
Flowchart of participant selection

Data spanning five NHANES cycles (2007–2016) were incorporated; these five cycles had full exposure, outcome, and covariate data, with 50,588 individuals across all cycles. Of these, 29,201 participants aged ≥ 20 years were initially considered. Individuals aged < 20 years and those lacking data on urinary PAH metabolites, hyperlipidemia status, or relevant covariates were not included, resulting in an analytical cohort of 7,030 subjects. The selection process is illustrated in the flow chart in Fig. 1.

### Urinary PAH metabolite Assessment

Urine specimens were procured by skilled technicians utilizing sterile polypropylene containers within the mobile examination center and subsequently stored at -20 °C in accordance with rigorous storage protocols before analysis at the National Center for Environmental Health. Analytical procedures entailed enzymatic hydrolysis of OH-PAH metabolites in urine, followed by their extraction via an online solid-phase extraction technique. Isotope dilution high-performance liquid chromatography-tandem mass spectrometry (online SPE-HPLC-MS/MS) facilitated the separation and quantification of these metabolites. The detailed protocols, analytical methods, and quality assurance measures used were sourced from NHANES official website. Between 2007 and 2016, the study analyzed five urinary PAH metabolites of low-molecular-weight (1-hydroxynaphthalene, 2-hydroxyfluorene, 2-hydroxynaphthalene, 1-hydroxyphenanthrene, and 3-hydroxyfluorene) alongside a urinary PAH metabolite of high-molecular-weight (1-hydroxypyrene), making up a total of six urinary metabolites under investigation. The limit of detection (LOD) is the minimum concentration at which an instrument can detect the presence or absence of an analyte. The LOD is typically determined under specific signal-to-noise ratio (S/N) conditions [20]. Urinary PAH metabolite concentrations were standardized to ng/L, with the LOD defined by an S/*N* ≥ 3 [12]. The detection rate for most urinary PAH metabolites surpassed 98%, while 1-hydroxypyrene was detected at a rate of 87.3%. The LODs and detection rates of the six urinary PAH metabolites are shown in Table S1. To account for urinary dilution variability, urinary PAH metabolite concentrations were creatinine-adjusted [21].

### Hyperlipidemia evaluation

Blood lipid metrics, encompassing fasting TGs, TC, LDL-C, and high-density lipoprotein cholesterol (HDL-C), underwent determination via lab analyses. Utilizing the Friedewald formula, LDL-C was estimated as LDL-C = TC - HDL-C - TG/5. Criteria for hyperlipidemia, as delineated by the Adult Treatment Panel III (ATP III) of the National Cholesterol Education Program (NCEP), included LDL-C ≥ 130 mg/dL, TC ≥ 200 mg/dL, TGs ≥ 150 mg/dL, or HDL-C ≤ 50 mg/dL in females and ≤ 40 mg/dL in males [22]. Participants who used antihyperlipidemic medications were also considered to have hyperlipidemia [23]. Fulfilment of any of these criteria confirmed a diagnosis of hyperlipidemia.

### Covariates

In this investigation, the analysis incorporated a comprehensive array of covariates, including age (categorized into three brackets: ≥ 20–39, 40–59, 60–80 + years), race (non-Hispanic Black, non-Hispanic White, Mexican American, other Hispanic, and other races), sex (female, male), poverty income ratio (PIR, stratified into < 1.30, 1.30–3.49, and ≥ 3.50), body mass index (BMI, categorized as < 25, 25–30, and > 30 kg/m²) [12], educational level (college or above, high school or GED and less than high school), smoking status (current smokers, former smokers and never smokers) [24], marital status (married, living with a partner, and living alone, which included widowed, divorced, separated, and never married), drinking status (drinkers and nondrinkers), physical activity (engagement in moderate-intensity recreational activities for at least 10 min weekly), hypertension (defined as systolic blood pressure ≥ 140 mmHg or diastolic blood pressure ≥ 90 mmHg, diagnosis by a health care professional, or current use of antihypertensive medication), and diabetes (defined by 2-hour oral glucose tolerance test (OGTT) glucose ≥ 11.1 mmol/L, fasting blood glucose ≥ 7.0 mmol/L, HbA1c > 6.5%, random blood glucose ≥ 11.1 mmol/L, use of insulin or diabetes medication, or self-reported diagnosis) [24]. These variables were derived from the 2007–2016 NHANES dataset.

### Statistical analysis

This study employed weighted methodologies to account for the intricate survey design inherent in NHANES data collection. Following the NHANES analysis protocols, weights for individual cycles were modified to account for the five cycles being analyzed. Continuous variables are showed as medians (interquartile ranges, IQRs) or means ± standard deviations (SDs), while categorical variables are described using unweighted counts and weighted percentages. Differences in groups of individuals with and without hyperlipidemia were assessed through the application of the weighted Student’s t test, Mann‒Whitney U test, and chi‒square test. The concentrations of the urinary PAH metabolites were categorized into quintiles (Q1-Q5) [25, 26]. Odds ratios (ORs) and 95% confidence intervals (CIs) were calculated using multivariable logistic regression analysis, investigating the relationship between levels of urinary PAH metabolites and hyperlipidemia through three distinct models: Model 1 without adjustments; Model 2 incorporated adjustments for demographic variables, including age, sex, and race; and Model 3, further refined to account for marital status, education, BMI, PIR, drinking and smoking behaviors, physical activity, and the presence of diabetes and hypertension. Utilizing a restricted cubic spline (RCS) methodology within Model 3, the study explored dose-response curves linking concentrations of urinary PAH metabolites to hyperlipidemia susceptibility. Continuous urinary PAH metabolite concentrations were log-transformed to ensure uniformity in the RCS analysis. To evaluate the impact of possible confounding variables on the relationship between urinary PAH metabolite levels and the prevalence of hyperlipidemia, analyses of subgroups and interactions were performed.

To evaluate the combined effects of multiple urinary PAH metabolites on hyperlipidemia, weighted quantile sum (WQS) regression was performed using the “gWQS” R package. WQS regression yields a unidirectional weighted index derived from quantitative data on chemical exposures, addressing dimensionality and multicollinearity in co-exposure scenarios [27]. The model generated two weighted indices for positive and negative effects to estimate the combined impact of all urinary PAH metabolites on hyperlipidemia. In addition, contributions to the overall effect were determined by calculating the relative weights of each exposure variable. In the WQS regression analysis, a random allocation assigned 40% of participants to a training set, while the remaining 60% formed the validation set. Statistical analyses were conducted in R studio (version 4.3.1), setting the threshold for statistical significance at *P* < 0.05.

## Results

### Participant characteristics

Participant characteristics are detailed in Table 1. The study sample consisted of 7,030 individuals aged ≥ 20 years, 71.5% of whom were diagnosed with hyperlipidemia. NHANES, employing a complex multistage probability sampling approach, utilizes weights to eliminate the bias caused by the sampling survey. After applying NHANES weights, the analysis encompassed a weighted total of 176,374,561 individuals. Based on the weighted results, the average participant age was calculated at 47.22 ± 16.73 years, 3,493 participants were female, and 3,537 participants were male. Individuals were categorized into two groups: hyperlipidemia and nonhyperlipidemia. For both groups, significant differences were observed in age, race, drinking status, marital status, smoking status, physical activity, BMI, educational level, diabetes mellitus, hypertension, and urinary PAH metabolite concentrations (*P* < 0.05).

**Table 1:** Characteristics related to demographics and health of participants in the NHANES 2007–2016

Characteristics	Total (*N* = 7,030)	Hyperlipidemia (*N* = 5,029)	Non-Hyperlipidemia (*N* = 2,001)	*P*-value
Age (years)	47.22 ± 16.73	50.13 ± 16.26	40.31 ± 15.79	< 0.001
PIR	3.00 ± 1.65	3.02 ± 1.64	2.93 ± 1.67	0.072
BMI (kg/m^2^)	29.03 ± 6.79	29.92 ± 6.75	26.90 ± 6.42	< 0.001
Age group (years), n (%)				< 0.001
≥20–39	2,433 (36%)	1,401 (28%)	1,032 (54%)	
40–59	2,322 (38%)	1,739 (41%)	583 (31%)	
60–80+	2,275 (25%)	1,889 (30%)	386 (14%)	
Gender, n (%)				0.500
Female	3,493 (51%)	2,535 (51%)	958 (50%)	
Male	3,537 (49%)	2,494 (49%)	1,043 (50%)	
Race, n (%)				0.002
Non-Hispanic White	3,100 (69%)	2,271 (70%)	829 (66%)	
Non-Hispanic Black	1,412 (11%)	941 (9.7%)	471 (13%)	
Mexican American	1,038 (8.1%)	755 (7.9%)	283 (8.4%)	
Other Hispanic	728 (5.5%)	542 (5.4%)	186 (5.6%)	
Other race	752 (7.1%)	520 (7.1%)	232 (7.1%)	
BMI group (kg/m^2^), n (%)				< 0.001
<25	2,030 (30%)	1,159 (23%)	871 (45%)	
25–30	2,351 (34%)	1,731 (35%)	620 (32%)	
>30	2,649 (36%)	2,139 (42%)	510 (23%)	
PIR group, n (%)				0.200
<1.30	2,272 (21%)	1,626 (21%)	646 (23%)	
1.30–3.49	2,575 (35%)	1,862 (36%)	713 (35%)	
≥3.50	2,183 (43%)	1,541 (44%)	642 (42%)	
Educational level, n (%)				< 0.001
Less than high school	1,660 (16%)	1,266 (17%)	394 (13%)	
High school or GED	1,595 (22%)	1,204 (24%)	391 (18%)	
College or above	3,775 (62%)	2,559 (59%)	1,216 (69%)	
Marital status, n (%)				< 0.001
Living alone	2,838 (37%)	1,935 (35%)	903 (42%)	
Living with a partner	582 (7.8%)	388 (7.1%)	194 (9.4%)	
Married	3,610 (55%)	2,706 (58%)	904 (49%)	
Smoking status, n (%)				< 0.001
Current smoker	1,461 (20%)	1,065 (20%)	396 (19%)	
Former smoker	1,714 (25%)	1,293 (27%)	421 (21%)	
Never smoker	3,855 (55%)	2,671 (53%)	1,184 (60%)	
Drinking status, n (%)				< 0.001
Yes	5,145 (78%)	3,617 (77%)	1,528 (81%)	
No	1,885 (22%)	1,412 (23%)	473 (19%)	
Physical activity, n (%)				< 0.001
Yes	3,378 (54%)	2,268 (51%)	1,110 (61%)	
No	3,652 (46%)	2,761 (49%)	891 (39%)	
Hypertension, n (%)				< 0.001
Yes	2,937 (37%)	2,396 (43%)	541 (23%)	
No	4,093 (63%)	2,633 (57%)	1,460 (77%)	
Diabetes, n (%)				< 0.001
Yes	1,309 (15%)	1,125 (18%)	184 (6.3%)	
No	5,721 (85%)	3,904 (82%)	1,817 (93.7%)	
1-hydroxynaphthalene	0.16 (0.08, 0.53)	0.17 (0.08, 0.59)	0.15 (0.07, 0.42)	< 0.001
2-hydroxynaphthalene	0.43 (0.22, 0.92)	0.44 (0.23, 0.95)	0.39 (0.21, 0.83)	0.002
3-hydroxyfluorene	0.01 (0.00, 0.02)	0.01 (0.00, 0.02)	0.01 (0.00, 0.02)	> 0.9
2-hydroxyfluorene	0.02 (0.01, 0.05)	0.02 (0.01, 0.05)	0.02 (0.01, 0.04)	0.014
1-hydroxyphenanthrene	0.013 (0.008, 0.021)	0.013 (0.008, 0.021)	0.012 (0.008, 0.020)	< 0.001
1-hydroxypyrene	0.012 (0.007, 0.021)	0.012 (0.007, 0.021)	0.012 (0.008, 0.022)	0.026
Ʃ PAHs	0.73 (0.42, 1.57)	0.76 (0.43, 1.67)	0.67 (0.40, 1.39)	< 0.001

Continuous variables are presented as median (interquartile range, IQR) or mean ± standard deviation (SD), while categorical variables are shown as unweighted counts (n) with weighted percentages (%). *Ʃ* *PAHs* combined total of all urinary PAH metabolites, *PAH* Polycyclic aromatic hydrocarbon (ng/g creatinine) * 0.01, *BMI* body mass index, *PIR* Poverty income ratio

### Associations between urinary PAH metabolite levels and hyperlipidemia risk by Multivariate models and dose–response analysis

**Fig. 2:**
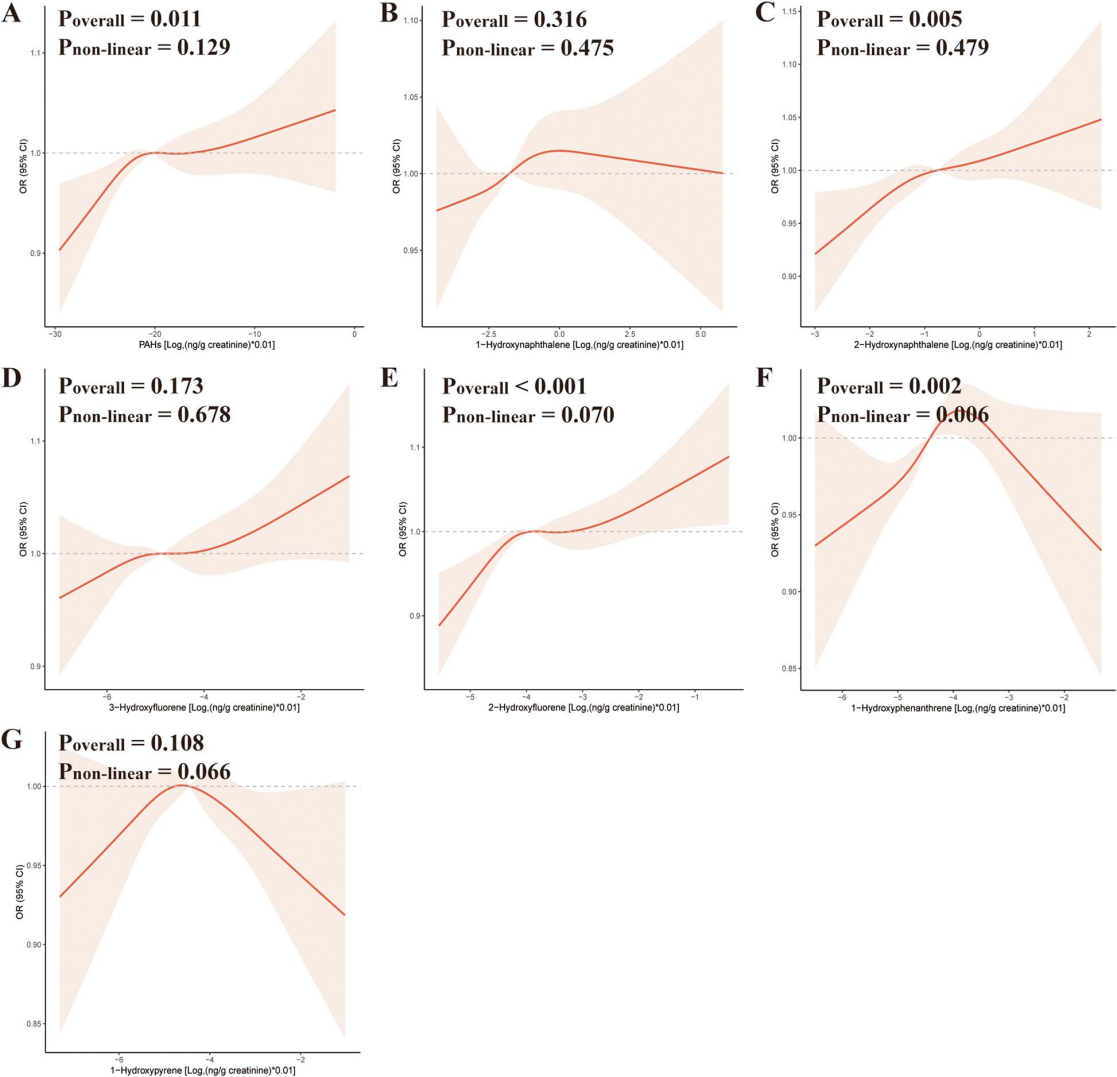
Dose–response relationships of urinary PAH metabolites with hyperlipidemia prevalence. Abbreviations: Ʃ PAHs, combined total of all urinary PAH metabolites; OR, odds ratio; CI, confidence interval. The models incorporated adjustments for several factors: sex, age, marital status, race, level of education, BMI, poverty income ratio (PIR), smoking and drinking behaviors, levels of physical activity, diabetes, and hypertension

The quantile-specific distributions of urinary PAH metabolite concentrations and the corresponding individual counts are presented in Table S2. Three distinct models were employed to examine relationships between urinary PAH metabolite levels and hyperlipidemia risk (Table 2). Initial unadjusted analyses (Model 1) indicated a significant increase in the prevalence of hyperlipidemia associated with Q4 and Q5 of urinary PAH metabolite concentrations (OR = 1.28, 95% CI: 1.07–1.54 and OR = 1.47, 95% CI: 1.21–1.80, respectively). Subsequent adjustment in Model 2 for demographic variables (age, race, and sex) further underscored this association, with ORs of 1.38 (95% CI: 1.13–1.69) and 1.50 (95% CI: 1.23–1.84) for Q4 and Q5, respectively. Comprehensive adjustments in Model 3 incorporating additional covariates such as education level, marital status, BMI, PIR, physical activity, drinking and smoking status, diabetes, and hypertension, revealed a persistently elevated risk within the highest quintile of urinary PAH metabolite exposure (OR = 1.33, 95% CI: 1.01–1.75). The RCS model demonstrated a significant monotonic dose-response correlation between levels of urinary PAH metabolites and the risk of hyperlipidemia (*P*-overall = 0.011, *P*-nonlinear = 0.129), as illustrated in Fig. 2.

**Table 2:** Urinary PAH metabolites levels associate with hyperlipidemia in the US adult population

ƩPAHs quintiles	Model_1		Model_2		Model_3	
	OR (95% CI)	*P*	OR (95% CI)	*P*	OR (95% CI)	*P*
Quintile_1	Reference		Reference		Reference	
Quintile_2	1.19 (0.94–1.51)	0.200	1.15 (0.90–1.48)	0.300	1.13 (0.87–1.48)	0.400
Quintile_3	1.10 (0.89–1.36)	0.400	1.08 (0.87–1.33)	0.500	1.05 (0.84–1.31)	0.700
Quintile_4	1.28 (1.07–1.54)	0.008	1.38 (1.13–1.69)	0.002	1.22 (0.96–1.56)	0.100
Quintile_5	1.47 (1.21–1.80)	< 0.001	1.50 (1.23–1.84)	< 0.001	1.33 (1.01–1.75)	0.044
P for trend		< 0.001		< 0.001		0.075

*PAH* Polycyclic aromatic hydrocarbon (ng/g creatinine) * 0.01, *Ʃ* *PAHs* combined total of all urinary PAH metabolites; odds ratio, and OR; confidence interval, and CI.

Logistic regression analysis was performed. Model_1 is without adjustments. Model_2 is modified to account for factors such as age, race, and sex. Model_3 incorporates adjustments for age, race, sex, BMI, marital status, educational attainment, poverty income ratio (PIR), drinking and smoking behaviors, diabetes, hypertension, and physical activity levels

### Relationships between six urinary PAH metabolites and hyperlipidemia: insights from logistic regression analysis

Table 3 displays the relationships between specific urinary PAH metabolites and hyperlipidemia. Logistic regression analyses revealed significant associations between higher quintiles of specific metabolites and hyperlipidemia prevalence in unadjusted models (Model 1). Notably, increased odds were detected for Q4 and Q5 of 2-hydroxynaphthalene (OR = 1.24, 95% CI: 1.02–1.51 and OR = 1.34, 95% CI: 1.07–1.68, respectively), the highest quintile of 2-hydroxyfluorene (OR = 1.28, 95% CI: 1.04–1.57), and for certain quintiles of 1-hydroxynaphthalene and 1-hydroxyphenanthrene.

**Table 3:** Urinary PAHs metabolite levels are associated with hyperlipidemia in the US adults

Urinary PAH metabolites (ng/g creatinine) * 0.01	Quintile_1		Quintile_2		Quintile_3		Quintile_4		Quintile_5		*P* for trend
	OR (95% CI)	*P*	OR (95% CI)	*P*	OR (95% CI)	*P*	OR (95% CI)	*P*	OR (95% CI)	*P*	
**1-Hydroxynaphthalene**											
Model_1	Reference		1.00 (0.77–1.31)	> 0.9	1.38 (1.13, 1.69)	0.002	1.08 (0.86, 1.37)	0.500	1.50 (1.19, 1.89)	< 0.001	< 0.001
Model_2	Reference		0.87 (0.67–1.13)	0.300	1.10 (0.90–1.35)	0.400	0.94 (0.74–1.19)	0.600	1.27 (1.01–1.60)	0.044	0.045
Model_3	Reference		0.95 (0.72–1.26)	0.700	1.28 (1.03–1.61)	0.030	1.00 (0.77–1.30)	> 0.9	1.32 (0.97–1.80)	0.072	0.131
**2-Hydroxynaphthalene**											
Model_1	Reference		1.07 (0.89–1.29)	0.500	1.11 (0.88–1.39)	0.400	1.24 (1.02–1.51)	0.032	1.34 (1.07–1.68)	0.012	0.002
Model_2	Reference		1.20 (0.99–1.45)	0.068	1.27 (1.00-1.60)	0.051	1.60 (1.33–1.93)	< 0.001	1.62 (1.30–2.03)	< 0.001	< 0.001
Model_3	Reference		1.18 (0.95–1.45)	0.130	1.16 (0.90–1.51)	0.200	1.35 (1.09–1.68)	0.007	1.31 (0.98–1.74)	0.065	0.022
**3-Hydroxyfluorene**											
Model_1	Reference		0.73 (0.58–0.93)	0.010	0.88 (0.70–1.11)	0.300	0.77 (0.61–0.97)	0.030	0.95 (0.76–1.18)	0.600	0.883
Model_2	Reference		0.77 (0.61–0.98)	0.032	0.89 (0.70–1.12)	0.300	0.89 (0.71–1.13)	0.300	1.15 (0.93–1.42)	0.200	0.068
Model_3	Reference		0.85 (0.67–1.07)	0.200	1.04 (0.82–1.32)	0.800	0.99 (0.77–1.26)	> 0.9	1.15 (0.87–1.53)	0.300	0.238
**2-Hydroxyfluorene**											
Model_1	Reference		1.09 (0.91–1.32)	0.300	1.19 (0.96–1.48)	0.100	1.12 (0.93–1.35)	0.200	1.28 (1.04–1.57)	0.020	0.028
Model_2	Reference		1.15 (0.93–1.40)	0.200	1.28 (1.01–1.61)	0.037	1.24 (1.02–1.52)	0.034	1.53 (1.23–1.91)	< 0.001	< 0.001
Model_3	Reference		1.19 (0.99–1.49)	0.130	1.33 (1.03–1.72)	0.031	1.24 (1.01–1.54)	0.044	1.39 (1.03–1.87)	0.033	0.010
**1-Hydroxyphenanthrene**											
Model_1	Reference		1.05 (0.85–1.30)	0.600	1.22 (1.02–1.46)	0.033	1.41 (1.16–1.72)	< 0.001	1.34 (1.12–1.59)	0.002	< 0.001
Model_2	Reference		1.05 (0.83–1.32)	0.700	1.19 (0.99–1.43)	0.065	1.36 (1.10–1.67)	0.005	1.22 (1.02–1.47)	0.031	0.004
Model_3	Reference		1.07 (0.84–1.37)	0.600	1.18 (0.98–1.43)	0.086	1.33 (1.06–1.67)	0.014	1.22 (0.99–1.51)	0.060	0.016
**1-Hydroxypyrene**											
Model_1	Reference		0.94 (0.74–1.19)	0.600	0.80 (0.65–0.98)	0.028	0.81 (0.65–1.01)	0.066	0.82 (0.65–1.02)	0.078	0.014
Model_2	Reference		1.12 (0.88–1.43)	0.400	0.95 (0.76–1.17)	0.600	1.07 (0.83–1.37)	0.600	1.06 (0.84–1.33)	0.600	0.891
Model_3	Reference		1.17 (0.90–1.51)	0.200	0.96 (0.77–1.21)	0.700	1.11 (0.85–1.47)	0.400	0.99 (0.76–1.30)	> 0.9	0.527

Polycyclic aromatic hydrocarbon (ng/g creatinine) * 0.01, PAH; odds ratio, and OR; confidence interval, and CI

Logistic regression analysis was performed. Model_1 is without adjustments. Model_2 is modified to account for factors such as age, race, and sex. Model_3 incorporates adjustments for age, race, sex, BMI, marital status, educational attainment, poverty income ratio (PIR), drinking and smoking behaviors, diabetes, hypertension, and physical activity levels

Adjustments for age, race, and sex (Model 2) further revealed positive correlations between higher exposure levels of specific urinary PAH metabolites and hyperlipidemia risk. Enhanced associations were particularly notable for the highest quintile of 2-hydroxynaphthalene (OR = 1.60, 95% CI: 1.33–1.93; OR = 1.62, 95% CI: 1.30–2.03), along with marked correlations for other metabolites across varying exposure levels, affirming a consistent pattern linking elevated urinary PAH metabolite concentrations with an increased hyperlipidemia risk.

After comprehensive adjustment for covariates, including age, race, sex, marital status, lifestyle factors, PIR, educational level, BMI, diabetes, and hypertension, a discernible association emerged between specific urinary PAH metabolites and hyperlipidemia. Specifically, Q4 of 2-hydroxynaphthalene exhibited a positive correlation with hyperlipidemia (OR = 1.35, 95% CI: 1.09–1.68; *P* for trend = 0.022; Model 3; Table 3). Similarly, Q3-Q5 of 2-hydroxyfluorene showed a correlation with elevated hyperlipidemia risk (OR = 1.33, 1.24, and 1.39; 95% CI: 1.03–1.72, 1.01–1.54, and 1.03–1.87; *P* for trend = 0.010; Model 3; Table 3), as was Q4 of 1-hydroxyphenanthrene (OR = 1.33, 95% CI: 1.06–1.67; *P* for trend = 0.016; Model 3; Table 3). Notably, the prevalence of hyperlipidemia showed no significant differences associated with 1-hydroxypyrene and 3-hydroxyfluorene concentrations (*P* > 0.05). The RCS model demonstrated linear dose‒response relationships for both 2-hydroxynaphthalene (*P*-overall = 0.005, *P*-nonlinear = 0.479) and 2-hydroxyfluorene (*P*-overall < 0.001, *P*-nonlinear = 0.070) with respect to the risk of hyperlipidemia, while an inverted U-shaped curve was detected between 1-hydroxyphenanthrene concentration and hyperlipidemia risk (*P*-overall = 0.002, *P*-nonlinear = 0.006), as depicted in Fig. 2.

### Associations between urinary PAH metabolite levels and hyperlipidemia across different subgroups

Subgroup analyses based on demographic and health-related characteristics revealed variable associations between urinary PAH metabolite levels and hyperlipidemia across different stratifications (Table 4, Table S3-S8). In participants aged 20–39 years, elevated levels of 3-hydroxyfluorene and 2-hydroxyfluorene were linked to a higher hyperlipidemia risk. Among those aged 60–80 + years, all levels of 1-hydroxynaphthalene and Q3-Q5 of 1-hydroxyphenanthrene showed a positive correlation to hyperlipidemia incidence. Sex-specific analysis indicated that Q3-Q5 of 1-hydroxyphenanthrene were positively associated with hyperlipidemia in females. Racial stratification revealed that Q4 and Q5 of 2-hydroxynaphthalene, along with Q3-Q5 of both 2-hydroxyfluorene and 1-hydroxyphenanthrene, were linked to an increased hyperlipidemia risk among non-Hispanic White subjects, while the highest levels of 3-hydroxyfluorene were positively associated with hyperlipidemia in other races. Drinking status revealed significant correlations between urinary PAH metabolites and Q4 and Q5 of 2-hydroxynaphthalene in drinkers. PIR-stratified findings indicated significant associations for the highest levels of 1-hydroxynaphthalene and 2-hydroxyfluorene within the PIR range of 1.30–3.49, while associations for 1-hydroxyphenanthrene were significant at a PIR < 1.30. Marital status analysis revealed that Q4 and Q5 of 2-hydroxynaphthalene and the highest levels of 3-hydroxyfluorene were positively correlated with hyperlipidemia among individuals living alone. Finally, educational level stratification revealed significant differences for all levels of 2-hydroxynaphthalene and for Q4 and Q5 of 1-hydroxyphenanthrene among participants with a college education or above.

**Table 4:** Stratifying variables reveal the association between urinary PAH metabolite levels and hyperlipidemia

	Quintile_1		Quintile_2		Quintile_3		Quintile_4		Quintile_5		*P* for interaction
	OR (95% CI)	*P*	OR (95% CI)	*P*	OR (95% CI)	*P*	OR (95% CI)	*P*	OR (95% CI)	*P*	
**Age group (years)**											0.064
≥ 20–39	Reference		1.25 (0.88–1.79)	0.2	0.91 (0.65–1.27)	0.6	1.25 (0.85–1.84)	0.3	1.61 (1.06–2.45)	0.026	
40–59	Reference		0.78 (0.48–1.26)	0.3	0.94 (0.59–1.50)	0.8	1.05 (0.65–1.72)	0.8	0.88 (0.51–1.52)	0.6	
60–80+	Reference		1.57 (0.98–2.51)	0.059	1.49 (0.92–2.40)	0.1	1.32 (0.83–2.09)	0.2	1.49 (0.83–2.67)	0.2	
**Gender**											0.072
Male	Reference		1.30 (0.91–1.85)	0.2	1.05 (0.75–1.45)	0.8	1.36 (0.96–1.91)	0.08	1.44 (0.99–2.09)	0.056	
Female	Reference		0.91 (0.62–1.33)	0.6	0.97 (0.67–1.41)	0.9	1.04 (0.72–1.51)	0.8	1.17 (0.80–1.71)	0.4	
**Race**											0.096
Mexican American	Reference		1.08 (0.59–1.95)	0.8	0.88 (0.44–1.76)	0.7	0.85 (0.45–1.64)	0.6	1.25 (0.58–2.71)	0.6	
Other Hispanic	Reference		1.46 (0.71–2.98)	0.3	0.58 (0.32–1.06)	0.076	0.87 (0.41–1.85)	0.7	0.82 (0.42–1.61)	0.6	
Non-Hispanic White	Reference		1.22 (0.83–1.79)	0.3	1.18 (0.85–1.62)	0.3	1.38 (0.96–1.98)	0.082	1.43 (0.98–2.10)	0.065	
Non-Hispanic Black	Reference		0.48 (0.34–0.66)	< 0.001	0.70 (0.46–1.05)	0.084	0.72 (0.51–1.02)	0.061	0.94 (0.63–1.42)	0.8	
Other/multiracial	Reference		1.49 (0.77–2.87)	0.2	0.90 (0.44–1.81)	0.8	1.86 (0.95–3.63)	0.069	0.97 (0.42–2.29)	> 0.9	
**Marital status**											0.175
Married	Reference		1.14 (0.81–1.60)	0.4	0.97 (0.72–1.30)	0.8	1.19 (0.86–1.64)	0.3	1.40 (0.91–2.15)	0.13	
Living with a partner	Reference		1.73 (0.75–3.99)	0.2	2.38 (1.25–4.54)	0.009	1.46 (0.72–2.99)	0.3	1.81 (0.78–4.21)	0.2	
Living alone	Reference		1.10 (0.71–1.71)	0.7	1.06 (0.75–1.50)	0.7	1.30 (0.87–1.95)	0.2	1.25 (0.79–1.98)	0.3	
**Educational level**											0.034
Less than high school	Reference		1.07 (0.61–1.88)	0.8	0.90 (0.52–1.55)	0.7	1.10 (0.67–1.81)	0.7	1.11 (0.66–1.86)	0.7	
High school or GED	Reference		0.45 (0.26–0.78)	0.005	0.58 (0.34-1.00)	0.049	0.54 (0.30–0.97)	0.041	0.88 (0.50–1.55)	0.7	
College or above	Reference		1.36 (1.00-1.86)	0.051	1.18 (0.91–1.53)	0.2	1.47 (1.10–1.96)	0.01	1.44 (1.00-2.08)	0.052	
**BMI group (kg/m**^**2**^**)**											0.192
< 25	Reference		0.96 (0.66–1.39)	0.8	1.00 (0.66–1.50)	> 0.9	1.47 (0.94–2.29)	0.087	1.55 (0.94–2.57)	0.087	
25–30	Reference		1.48 (0.93–2.36)	0.1	0.95 (0.63–1.44)	0.8	1.28 (0.80–2.03)	0.3	1.13 (0.68–1.89)	0.6	
> 30	Reference		1.06 (0.66–1.70)	0.8	1.20 (0.78–1.87)	0.4	1.01 (0.64–1.61)	> 0.9	1.36 (0.82–2.27)	0.2	
**PIR**											0.424
< 1.30	Reference		0.97 (0.62–1.52)	0.9	0.80 (0.52–1.22)	0.3	0.95 (0.61–1.47)	0.8	1.04 (0.64–1.69)	0.9	
1.30–3.49	Reference		1.32 (0.90–1.94)	0.15	1.20 (0.81–1.79)	0.4	1.41 (0.95–2.08)	0.086	1.34 (0.90–1.98)	0.15	
≥ 3.50	Reference		1.08 (0.72–1.63)	0.7	1.05 (0.74–1.50)	0.8	1.19 (0.78–1.82)	0.4	1.54 (0.85–2.77)	0.2	
**Smoking status**											0.605
Never smoker	Reference		1.06 (0.78–1.44)	0.7	0.92 (0.71–1.20)	0.6	1.15 (0.85–1.55)	0.4	1.03 (0.68–1.57)	0.9	
Former smoker	Reference		1.37 (0.86–2.18)	0.2	1.57 (0.97–2.54)	0.066	1.59 (0.93–2.73)	0.089	1.67 (0.92–3.05)	0.091	
Current smoker	Reference		2.13 (0.31–14.5)	0.4	0.92 (0.17–5.13)	> 0.9	1.27 (0.24–6.74)	0.8	1.60 (0.31–8.39)	0.6	
**Drinking status**											0.806
Yes	Reference		1.22 (0.89–1.67)	0.2	1.17 (0.90–1.51)	0.2	1.32 (1.00-1.74)	0.054	1.47 (1.08-2.00)	0.016	
No	Reference		0.90 (0.57–1.43)	0.7	0.71 (0.45–1.11)	0.13	0.94 (0.61–1.44)	0.8	0.94 (0.53–1.66)	0.8	
**Physical activity**											0.911
Yes	Reference		1.19 (0.86–1.65)	0.3	1.15 (0.84–1.56)	0.4	1.14 (0.81–1.59)	0.4	1.29 (0.89–1.87)	0.2	
No	Reference		1.04 (0.72–1.50)	0.8	0.86 (0.59–1.26)	0.4	1.27 (0.88–1.83)	0.2	1.32 (0.82–2.14)	0.2	
**Hypertension**											0.963
Yes	Reference		1.02 (0.68–1.52)	> 0.9	1.24 (0.80–1.90)	0.3	1.10 (0.69–1.75)	0.7	1.26 (0.75–2.12)	0.4	
No	Reference		1.18 (0.86–1.63)	0.3	0.98 (0.76–1.28)	> 0.9	1.26 (0.93–1.72)	0.14	1.30 (0.96–1.76)	0.087	
**Diabetes**											0.942
Yes	Reference		1.06 (0.54–2.10)	0.9	1.35 (0.72–2.53)	0.3	1.45 (0.77–2.74)	0.2	2.07 (0.97–4.41)	0.06	
No	Reference		1.14 (0.87–1.50)	0.3	1.02 (0.81–1.28)	0.9	1.19 (0.91–1.54)	0.2	1.26 (0.94–1.69)	0.13	

Polycyclic aromatic hydrocarbon (ng/g creatinine) * 0.01, PAH. The first quintile of urinary PAH metabolites serves as the reference category. Stratification was conducted through logistic regression analysis. Adjust for age, race, sex, educational level, PIR, marital status, BMI, drinking status, physical activity, smoking status, diabetes, and hypertension

Additionally, an interaction analysis aimed to examine the impact of diverse demographic and clinical factors on the relationships between urinary PAH metabolite levels and hyperlipidemia (Table 4, Table S3-S8). A significant interaction was noted between urinary PAH metabolites, 1-hydroxynaphthalene, and education level. In addition, 1-hydroxynaphthalene showed a significant interaction with age. Furthermore, an interaction between 2-hydroxynaphthalene and smoking status was observed. Significant interactions were also found between 3-hydroxyfluorene, 1-hydroxyphenanthrene, and 2-hydroxyfluorene and alcohol consumption status. Moreover, 2-hydroxynaphthalene and 3-hydroxyfluorene interacted with diabetes. Additionally, a significant interaction between 1-hydroxyphenanthrene and PIR was identified (all *P* values for interactions < 0.05).

### WQS regression of the associations between urinary PAH metabolite co-exposure and hyperlipidemia

**Fig. 3:**
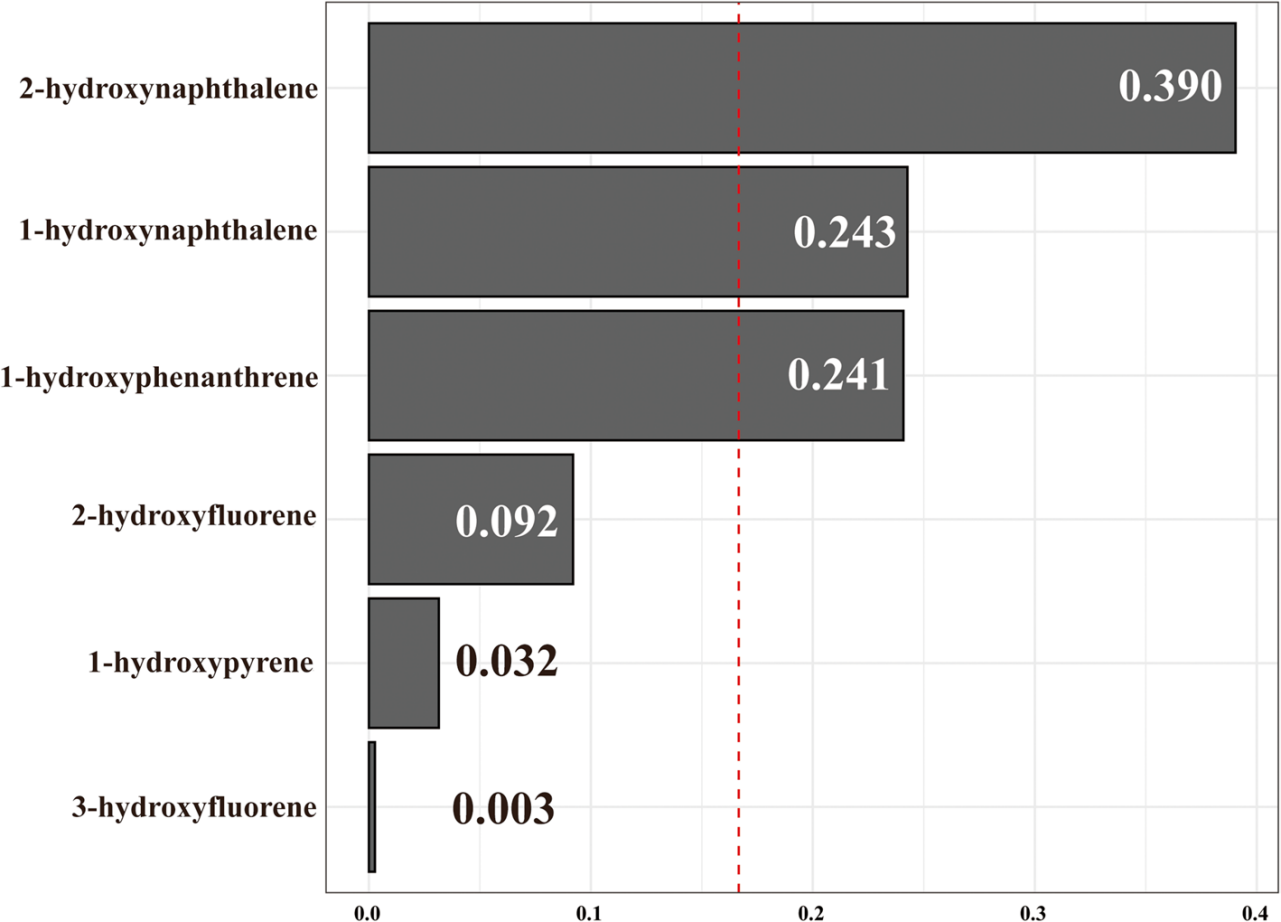
Combined association between urinary PAH metabolites and hyperlipidemia using the WQS model. Notes: To minimize skewness in the distribution of concentrations, all urinary PAH metabolites underwent log_2_ transformation. Abbreviations: WQS, weighted quantile sum

The WQS regression models were performed to access both the positive and negative directions. Following adjustments for all covariates, the results of the positive WQS regression showed positive correlations between urinary PAH metabolite mixtures and hyperlipidemia prevalence (OR = 1.04, 95% CI: 1.00-1.09, *P* < 0.05) (Table S9). 2-hydroxynaphthalene had the greatest impact on the risk of hyperlipidemia at 0.390, followed by 1-hydroxynaphthalene (0.243) and 1-hydroxyphenanthrene (0.241), indicating that these compounds play important roles in hyperlipidemia (Fig. 3). The WQS regression in the negative direction did not show any significant association between urinary PAH metabolite mixtures and hyperlipidemia prevalence (OR = 1.02, 95% CI: 0.98–1.06, *P* = 0.29), as shown in Table S9.

## Discussion

### Summary of the main results

This cross-sectional analysis investigated the impact of exposure to urinary PAH metabolites on hyperlipidemia among US adults. Adjustments for various covariates revealed a significant link between elevated urinary PAH metabolite concentrations and increased hyperlipidemia risk. Additionally, increased levels of urinary 2-hydroxynaphthalene, 1-hydroxyphenanthrene, and 2-hydroxyfluorene showed a clear positive correlation with hyperlipidemia prevalence. These findings indicate a relationship between exposure to urinary PAH metabolites and an elevated risk for hyperlipidemia. Subgroup analyses revealed positive associations between certain urinary PAH metabolites and hyperlipidemia prevalence among certain groups, such as individuals aged 20–39 years and 60–80 + years, females, non-Hispanic White subjects, drinkers, individuals with lower PIRs, those living alone, and those with a college degree or above. Moreover, the study highlighted positive dose-response relationships for 2-hydroxynaphthalene and 2-hydroxyfluorene with an increased prevalence of hyperlipidemia. The WQS model underscored a positive correlation between mixtures of urinary PAH metabolites and hyperlipidemia risk; notably, 2-hydroxynaphthalene was the most significant contributor. Thus, regulating atmospheric PAHs and reducing exposure to these compounds could be effective strategies for reducing the risk of hyperlipidemia and preventing its occurrence.

### Comparison with previous studies

PAHs, which are globally prevalent environmental pollutants, have attracted considerable attention because of their health hazards. The high prevalence of hyperlipidemia in the US, which is associated with various clinical metabolic disorders and cardiovascular diseases, imposes a significant burden on the global health care system [28]. Moreover, PAHs can affect metabolic processes in the body, such as obesity progression, adipocyte proliferation, and TC changes, and transfer to breast milk and the placenta, which may increase the risk of infant exposure [29, 30]. Recent epidemiological evidence has illuminated the effects of urinary PAH metabolites on lipid concentrations. For example, urinary PAH metabolites were positively associated with LDL-C and TC levels in a Wuhan-Zhuhai cohort study [16]. Ranjbar et al. demonstrated that higher levels of 1-naphthalene, 2-phenanthrene, 2-naphthalene, 2-fluorene, and 3-fluorene were positively associated with dyslipidemia risk in 4,675 adults in the US [25]. Wang et al. reported positive correlations between 2-naphthalene, 4-phenanthrene, and 9-fuorene and the percentage of trunk fat mass [31]. Moreover, Yang et al. reported that 1-hydroxynaphthalen, 2-hydroxynaphthalene, 1-hydroxyphenanthrene, and 2-hydroxyphenanthrene were positively associated with metabolic syndrome [9]. WQS analysis revealed that increased exposure to PAH mixtures was associated with a higher prevalence of metabolic syndrome, increased waist circumference, increased TGs, and decreased HDL-C [9]. Animal studies have shown that PAH exposure can elevate blood lipid levels by disrupting lipid metabolism in mice [18].

However, conflicting results have been reported by other studies. In an NHANES survey of 1,878 US nondiabetic adults from 2001 to 2008, Hu et al. reported negative correlations between 3-hydroxyfluorene, 1-hydroxypyrene, and TG levels, with no significant differences between hydroxyphenanthrene, hydroxynaphthalene and TG [19]. Another study reported no significant association between 1-phenanthrene and dyslipidemia risk [25]. Additionally, a Swedish cross-sectional investigation revealed no link between urinary PAH metabolites and HDL-C or TC concentrations [32].

### Potential biological mechanisms

This thorough analysis revealed several potential mechanisms by which urinary PAH metabolites contribute to an increased prevalence of hyperlipidemia. PAHs are distributed throughout various tissues and organs of the human body and exhibit high lipophilicity [10]. PAHs can influence lipid metabolism pathways by activating estrogen receptors and inhibiting thyroid receptors, leading to an increase in adipose tissue mass [33, 34]. Substances such as naphthalene, fluorene, and pyrene can affect adipocytes by activating estrogen-related genes, subsequently altering metabolism and lipid balance [35]. Furthermore, an animal study revealed that PAH metabolites could impede lipolysis in adipose tissue, leading to increased adiposity and body weight in mice [36]. Another study revealed that 2-hydroxynaphthalene contributed the most to hyperlipidemia among mixtures of urinary PAH metabolites. A potential explanation for the relationship between 2-hydroxynaphthalene and hyperlipidemia can be found in research conducted by Bright and Mlyczyńska et al. [37, 38]. In a study by Bright et al., 2-naphthol significantly promoted lipid accumulation in adipocytes by upregulating the expression levels of key markers associated with lipogenesis (CCAAT enhancer binding protein α and peroxisome proliferator-activated receptor gamma) and adipogenesis (fatty acid synthase) while concurrently downregulating the expression levels of markers of lipolysis (hormone-sensitive lipase and adipocyte triglyceride lipase) [37]. Mlyczyńska et al. reported that exposure to naphthalene led to enhanced cell proliferation and adipogenesis in 3T3-L1 preadipocytes, along with an upregulation of adipogenesis-related gene expression following cell differentiation [38]. These findings underscore the intricate mechanisms by which PAH exposure can disrupt lipid metabolism processes, ultimately contributing to the development of hyperlipidemia.

Previous research has indicated that the fundamental mechanism by which PAH metabolites induce hyperlipidemia may be linked to oxidative stress. PAH metabolites can activate the aryl hydrocarbon receptor (AhR) signaling pathway, facilitating their metabolism via cytochrome P450, and causing the formation of harmful reactive oxygen species (ROS) [6, 39]. The presence of PAH metabolites and excess ROS can initiate lipid oxidation and metabolic disturbances, culminating in abnormal cellular function and dyslipidemia [18, 40]. Furthermore, inflammatory responses have been proposed as contributing factors. Studies suggest that adipose tissue can store metabolites such as PAHs and inflammatory cytokines [41]. PAH metabolites trigger the release of significant quantities of proinflammatory cytokines from white adipose tissue and immune cells through activation of inflammatory pathways [37, 42]. The release of these inflammatory mediators not only induces an inflammatory response in local tissues but also impacts the overall metabolic equilibrium of the body. Specifically, inflammatory factors, such as TNF-α and IL-6, are associated with dyslipidemia. TNF-α is positively associated with LDL-C levels and inversely with HDL-C, whereas IL-6 correlates positively with TGs levels and inversely with HDL-C [43, 44]. Moreover, inflammation contributes to vascular endothelial dysfunction and disrupts the balance between lipid generation and breakdown, leading to increased lipid levels [45].

These observations revealed intriguing variations in the correlations between urinary PAH metabolite levels and hyperlipidemia across specific population characteristics. Notably, the link between levels of urinary PAH metabolites and hyperlipidemia was stronger in individuals aged 20–39 years and those with lower income levels, potentially because of unfavorable living conditions and unhealthy lifestyles and dietary practices within this demographic [46]. A more pronounced correlation was noted between urinary PAH metabolite levels and hyperlipidemia in females than in males, possibly due to the structural similarity of PAHs to estrogen and their estrogenic properties, suggesting a disruption of estrogen-mediated lipid-lowering effects by PAHs [31, 35]. Moreover, individuals with higher educational levels demonstrated stronger correlations between urinary PAH metabolite levels and hyperlipidemia, likely because they have the autonomy to select their living environments, often residing in urban or industrialized areas with heightened environmental pollution levels, including PAH exposure. Furthermore, the relationships between behavioral aspects, like alcohol intake, and hyperlipidemia highlight the critical role of lifestyle modifications in preventing and managing hyperlipidemia. The findings also highlighted a race-specific association between urinary PAH metabolite levels and hyperlipidemia, with a positive correlation in non-Hispanic White subjects within fully adjusted models, whereas no significant association was observed in other subgroups. The underlying reasons for this discrepancy, whether linked to lifestyle behaviors or living environments, warrant further investigation to elucidate the mechanisms driving this phenomenon.

### Strengths and limitations

Several strengths of this study added to the robustness of the findings. First, this investigation marks the pioneering effort to examine the association between levels of urinary PAH metabolites and hyperlipidemia within the US adults. Second, the study is grounded in the NHANES, aimed at offering data from a survey representative of the national population, thereby enhancing the potential generalizability of these results to the broader US population. Third, by utilizing urinary PAH metabolites as a measure for estimating PAH exposure, the researchers captured exposure data from diverse sources. Finally, the analysis investigated the impact of urinary PAH metabolites on hyperlipidemia prevalence across various subgroups while considering potential confounding factors. This research used the WQS model to comprehensively evaluate associations between urinary PAH metabolite mixtures and the risk of hyperlipidemia, and the weights of individual urinary PAH metabolites in the combined effect were assessed.

Despite the valuable insights provided by this research regarding the link of urinary PAH metabolites with hyperlipidemia, certain limitations should be acknowledged. First, the cross-sectional design of this research poses challenges in establishing causality. Second, despite the researchers’ efforts to adjust for numerous potential confounders, other variables that could have influenced the results may have been overlooked. Future studies should use a prospective cohort design to elucidate the causal link between PAH exposure and hyperlipidemia. Additionally, a more detailed exploration of the specific mechanisms through which different PAHs and their metabolites affect lipid metabolism will enhance the understanding of how environmental pollutants increase the hyperlipidemia risk. Furthermore, since urinary PAH metabolite concentrations do not directly reflect PAH levels stored in adipose tissue, further investigations are warranted to explore PAH levels in adipose tissue owing to their association with long-term health implications.

## Conclusion

This research revealed a significant positive correlation between urinary PAH metabolite concentrations and hyperlipidemia risk. Notably, 2-hydroxynaphthalene was the most influential among these urinary PAH metabolite mixtures. This discovery enhances the understanding of the link between environmental pollution and susceptibility to hyperlipidemia, suggests that urinary PAH metabolites may be a predictor of hyperlipidemia, which can help clinicians identify individuals at high risk for hyperlipidemia, and underscores the significance of mitigating PAH exposure to prevent hyperlipidemia and related cardiovascular conditions. Despite certain constraints, the findings lay solid groundwork for future investigations and offer crucial insights for public health interventions.

### Supplementary Information

Additional file 1: Table S1. Detection rates of 6 urinary PAHs metabolites; Table S2. Information of each quartile for different urinary PAHs metabolites; Table S3. Stratified Variables Illuminate the Association of 1-Hydroxynaphthalene with Hyperlipidemia in the US Population; Table S4. Stratified Variables Illuminate the Association of 2-Hydroxynaphthalene with Hyperlipidemia in the US Population; Table S5. Stratified Variables Illuminate the Association of 3-Hydroxyfluorene with Hyperlipidemia in the US Population; Table S6. Stratified Variables Illuminate the Association of 2-Hydroxyfluorene with Hyperlipidemia in the US Population; Table S7. Stratified Variables Illuminate the Association of 1-Hydroxyphenanthrene with Hyperlipidemia in the US Population; Table S8. Stratified Variables Illuminate the Association of 1-Hydroxypyrene with Hyperlipidemia in the US Population; Table S9. The joint effect of urinary PAHs metabolites mixtures on the prevalence of hyperlipidemia in WQS model in positive and negative direction.

## Data Availability

No datasets were generated or analysed during the current study.

## Abbreviations

PAHs: Polycyclic aromatic hydrocarbons
NHANES: National health and nutrition examination survey
TGs: Triglycerides
TC: Total cholesterol
LDL-C: Low-density lipoprotein cholesterol
HDL-C: High-density lipoprotein cholesterol
OH-PAHs: Mono-hydroxylated polycyclic aromatic hydrocarbons
NCHS: National center for health statistics
PIR: Poverty income ratio
CDC: Centers for disease control and prevention
BMI: Body mass index
OR: Odds ratio
CI: Confidence interval
RCS: Restricted cubic spline
WQS: Weighted quantile sum
LOD: Limit of detection
ROS: Reactive oxygen species
